# Repetition in social contacts: implications in modelling the transmission of respiratory infectious diseases in pre-pandemic and pandemic settings

**DOI:** 10.1098/rspb.2024.1296

**Published:** 2024-07-24

**Authors:** Neilshan Loedy, Jacco Wallinga, Niel Hens, Andrea Torneri

**Affiliations:** ^1^ Data Science Institute, Hasselt University, Hasselt, Belgium; ^2^ Center for Infectious Disease Control, National Institute for Public Health and the Environment, Blithoven, The Netherlands; ^3^ Department of Biomedical Data Sciences, Leiden University Medical Center, Leiden, The Netherlands; ^4^ Centre for Health Economics Research and Modelling Infectious Diseases, Vaccine & Infectious Disease Institute, University of Antwerp, Antwerp, Belgium

**Keywords:** transmission dynamics, epidemic models, social contact

## Abstract

The spread of viral respiratory infections is intricately linked to human interactions, and this relationship can be characterized and modelled using social contact data. However, many analyses tend to overlook the recurrent nature of these contacts. To bridge this gap, we undertake the task of describing individuals’ contact patterns over time by characterizing the interactions made with distinct individuals during a week. Moreover, we gauge the implications of this temporal reconstruction on disease transmission by juxtaposing it with the assumption of random mixing over time. This involves the development of an age-structured individual-based model, using social contact data from a pre-pandemic scenario (the POLYMOD study) and a pandemic setting (the Belgian CoMix study), respectively. We found that accounting for the frequency of contacts impacts the number of new, distinct, contacts, revealing a lower total count than a naive approach, where contact repetition is neglected. As a consequence, failing to account for the repetition of contacts can result in an underestimation of the transmission probability given a contact, potentially leading to inaccurate conclusions when using mathematical models for disease control. We, therefore, underscore the necessity of acknowledging contact repetition when formulating effective public health strategies.

## 1. Background

The propagation of infectious diseases is characterized by various modes of transmission. Transfer of pathogenic agents can occur through contact with fomites, vectors (e.g. insects or animals), airborne aerosols, droplets or direct physical contact [1]. Notably, viral respiratory infections, primarily transmitted during social interactions via droplets, aerosols and physical contact, continue to be major contributors to global morbidity and mortality [2]. Recognizing the significance of information on close interactions is pivotal for elucidating disease transmission [3]; thus, social contact data are often employed as a valuable proxy for identifying transmission routes. This information has primarily been collected through diary-based social contact surveys in various countries [4]. An important study is the groundbreaking POLYMOD study which is the first large-scale study that gathered data from eight countries in the European Union [5]. Numerous studies similar in set-up have been conducted in different countries in both pre-pandemic and pandemic periods [4]. The CoMix study is the largest study across Europe gathering social contact data aiming to assess changes in contact behaviour during the pandemic [2,6–11]. The information from these surveys has proved invaluable in informing transmission dynamics within mathematical models including network models [12], agent-based models [13] and compartmental models [14–16].

In contrast to our everyday experience, many mathematical models commonly assume a homogeneous mixing [17]. However, research demonstrates that mixing patterns exhibit heterogeneity influenced by factors like age, location, household sizes, intimacy levels and frequency of contact [5]. Incorporating this heterogeneity in mathematical models is therefore essential to accurately describe transmission dynamics. Various techniques have been developed to achieve this by using social contact data [18]. These methods often involve the use of contact matrices, which consider different contact rates for different subgroups of individuals within a structured population. The contact rate is typically calculated using the average number of contacts reported on a daily basis [19], implicitly assuming that the probability of contacting the same person more than once is negligible, and the time between successive contacts within a given age bracket follows an exponential distribution. This contrasts with our daily experience, where we, e.g. frequently interact with household members, a significant aspect of mixing patterns that is not entirely accounted for by modelling methods relying on contact matrices.

Eames *et al.* [20] and Smieszek *et al.* [21] have demonstrated the importance of considering contact repetition in representing disease transmission [20,21]. Their studies reveal that contact repetition can limit the spread of infection, significantly impacting epidemiological measures when the number of contacts and probability of transmissions are low, as opposed to a random mixing model. However, these studies primarily focused on the theoretical framework and exploration of repetition in mixing patterns using data that have not been fully realized. More recently, Pung *et al.* [22] also pointed out the importance of considering the temporal components of contact patterns, highlighting the implication of contact retention by temporally reconstructing dynamic contact networks using data collected through close proximity sensors [22]. While the use of sensor data provides advantages, the analysis conducted is reliant on information from specific locations and may not comprehensively capture real-life contacts typical in open populations.

Building on previous research, our objective is to reconstruct temporal contact patterns of subgroups within the population and calculate the age-specific number of distinct contacts over a one week period. We use social contact data obtained from contact diary surveys [2,5], exploring various age groups and different countries to elucidate contact patterns in various settings. Furthermore, we also consider the effect of non-pharmaceutical interventions, delineating how these temporal patterns fluctuated during the COVID-19 pandemic. With the reconstructed patterns, we developed an individual-based model to assess the influence of considering contact repetition, obtained from temporal contact reconstruction.

## 2. Methods

### (a) Temporal reconstructions of individuals’ contact patterns

Drawing from data obtained through the POLYMOD and Belgian CoMix studies [2,5], we reconstructed individuals’ contact patterns over a, without loss of generality, one week period to replicate the social contact dynamics of each individual. In both surveys, participants were asked to provide at least basic sociodemographic information and report contacts made on a day between 05.00 on the day preceding the survey day and 05.00 of the survey day. A contact was defined as either skin-to-skin contact such as a kiss or handshake (a physical contact) or a two-way conversation with three or more words in the physical presence of another person but no skin-to-skin contact (a nonphysical contact). Additionally, they were asked to provide supplementary information about these contacts. This included details such as age and the gender of the contacted person, whether the contact included skin-to-skin touching (physical/non-physical contact), the duration of the contact (less than 5 min, 5−15 min, 15 min to 1 h, 1−4 h or 4 h or more), the frequency (daily, weekly, monthly, a few times a year or for the first time) with which they usually contact this person and location of contacts (home, work, school, leisure activities and other places). In our main analysis, ‘contact’ is defined as conversational and non-conversational encounters involving skin-to-skin touching, which serves as a proxy for potential transmission events. We provide results from the analyses of physical contacts in the main manuscript to enhance clarity and comprehension for the reader, while comprehensive findings encompassing all contacts (both physical and non-physical) are available in the electronic supplementary material.

Using information from the POLYMOD study, we first scrutinized mixing patterns within a pre-pandemic setting. Subsequently, we consider a pandemic scenario relying on the data collected within the Belgian CoMix study. Unlike the POLYMOD study, which is a cross-sectional survey, the CoMix study employs a biweekly longitudinal approach. The longitudinal design of this survey has been observed to be susceptible to participant fatigue, a phenomenon that may occur when participants become bored or less interested in the survey and that can result in contacts underreporting [2]. While statistical methods for under-reporting have been previously developed for the total number of contacts, we have extended such methodology to also account for the frequency of contacts. This involved developing generalized additive models for location, scale and shape to estimate the number of contacts reported by each participant. We then used the model to predict the number of contacts that would have been reported if the survey had been taken for the first time and redistributed the estimated contacts using the age-specific contact frequency obtained from the data. The choice of the Belgian CoMix study was motivated by its extensive data collection period, allowing us to observe the impact of interventions on reported contacts over an extended time frame. Notably, the initial eight waves of the Belgian CoMix study lacked information on participants younger than 9 years old, prompting our focus on the later waves. Details regarding the used datasets are presented in the electronic supplementary material.

We divided the population into four age categories: children (0–12 years), teens (13–18 years), adults (19–65 years) and the elderly (66+years) and reconstructed temporal contact patterns for each of these subgroups. We did so by considering two approaches, a so-called naive approach, where only the number of contacts reported on a daily basis is considered and no frequency is accounted for, and the frequency-based approach. In the latter case, we focused on using the reported frequency of contacts, which indicates how often participants interacted with those they contacted, to reconstruct individuals’ contact patterns over a week. We categorized these interactions into two groups: ‘daily contacts’ (reported as occurring daily in the survey), involving interactions with the same individuals every day and ‘non-daily contacts’ (reported as weekly, monthly, a few times a year or for the first time in the survey)—encompassing interactions with different individuals throughout the week. Subsequently, we redistribute the number of distinct contacts over d=7 days using a multinomial distribution, where d is an index over days in the week (Monday, Tuesday, …, Sunday). The probabilities for this redistribution are derived from the data, acknowledging that individuals may have varying numbers of distinct contacts each day throughout the week. We assume that contacts for each individual follow a generalized Poisson distribution, allowing us to account for both under- and over-dispersion in the data and thereby capture contact variability [23,24]. We define k as an index representing the frequency of contact, partitioned into five categories (daily, weekly, monthly, a few times a year and first time) and a serves as an index for age groups (0–12, 13–18, 19–65 and 66+). The total number of distinct contacts of an individual i made in d day for a frequency of contact k is denoted by $\;\lambda_{k,i}^{*}$. Let $\mu_{a,k}$ and $\sigma_{a,k}^{2}$ be the mean and variance, respectively, for age group a with contact frequency k. Mathematically, the frequency-based approach can be expressed as:


$$\begin{array}{c} \lambda_{k,i}^{*}\left\{ \begin{array}{l} \lambda_{k,i}^{*}∼GPO\left(1,\alpha_{a,k},\;\beta_{a,k}\right),k=daily\\ \lambda_{k,i}^{*}∼GPO\left(d,\alpha_{a,k},\;\beta_{a,k}\right),k=weekly,monthly,a\;few\;times\;a\;year\;and\;first\;time \end{array}\right. \end{array}$$

with $\alpha_{a,k}=\mu_{a,k}\sqrt{\mu_{a,k}/\sigma_{a,k}^{2}}$ and $\beta_{a,k}=1-\sqrt{\mu_{a,k}/\sigma_{a,k}^{2}}$. An example showing the outcome of the frequency-based reconstruction is presented in the electronic supplementary material, table S1. Relying on this framework and the considered approaches to reconstruct contact patterns, the total number of distinct contacts in a week is calculated as $\lambda_{T,i}=\Sigma_{d=1}^{7}\lambda_{d,i}\;and\;\lambda_{T,i}=\lambda_{k=daily,i}+\Sigma_{d\;=\;1}^{7}\Sigma_{k\;=\;2}^{5}\lambda_{k\;\neq daily,d,i}$ for the naive and frequency-based approaches, respectively.

### (b) Epidemic simulation and scenarios

To investigate the impact of temporal contact patterns on transmission dynamics, we developed a discrete-time age-structured stochastic individual-based model in which contact patterns are assumed to represent plausible transmission pathways for influenza-like and COVID-19-like diseases implemented using either the naive or frequency-based approaches described previously. In both cases, epidemics start with seeding one index case in a closed and fully susceptible population and will persist until no infected individuals are present [25]. We simulated contact interactions for each infected individual, and we conducted a Bernoulli trial with probability q to assess whether the specific interaction led to a transmission event. Within both approaches, such a probability is constant and of the same value among the different age groups, but it is set to describe a different value of the reproduction number according to the social contact hypothesis [19]. In the naive approach, at each time step, every infected individual comes into contact with $c_{i}$ individuals drawn randomly from the population, setting the within and between mixing rate among the different age groups from the observed data. In the frequency-based approach, for each individual i, we randomly selected daily contacts, with whom i would have an interaction every day, and non-daily contacts, sampling different individuals who were not part of the daily interactions. In addition, we investigated the impact of contact reconstruction in different locations (inside and outside the house), the effect of the infection period on disease transmission and the effect of accounting for contact repetition on disease spread with the same number of reproductions between the two approaches (electronic supplementary material).

We consider a susceptible-infected-recovered (S-I-R) type of dynamics [26], assuming the infectious period to be geometrically distributed I∼Geo(γ) with an average of 7 days. We further set the values of the reproduction number, *R*_0_ = 1.3, to represent diseases with the spreading potential of a seasonal influenza-like illness [27] and *R*_0_ = 3.3, reflecting diseases with the spreading potential of a COVID-19-like infection [28]. Fixing the population size at *N* = 5000, we ran 3000 simulations for each considered scenario. From the simulated epidemics, we only considered the non-extinct outbreaks, defined as the outbreaks where at least 10% of the population was eventually infected, and we calculated and reported attack rates, time to peak, peak incidence and epidemic durations as epidemiological summary measures.

The simulations were run for all eight countries participating in the POLYMOD study and three different waves of the CoMix study for Belgium. These three different waves are wave 19, wave 27 and wave 43 (electronic supplementary material, table S2). These waves represent three distinct stages in the progression of interventions throughout the COVID-19 pandemic. Wave 19 identified a period where severe restrictions were in place. Measures during this wave include mandatory telework and mask-wearing, a curfew, part-time distance learning in schools, allowance for a maximum of 1 (or 2 for individuals living alone) close contact and the full closure (except for takeaway services) of restaurants, cafes and bars. Wave 27 represents a period where moderate restrictions were in place. In this period, the restrictions consisted of mandatory mask-wearing, compulsory telework and a limit of eight people per table for dining in restaurants, cafes and bars. Finally, in wave 43, there were only mild measures that included mandatory mask-wearing and the implementation of a COVID Safe Ticket system, which required individuals to provide proof of vaccination, recent recovery from COVID-19 or a negative test result to access certain venues or events [2].

## 3. Results

### (a) Pre-pandemic setting

#### (i) Contact repetition

**Figure 1.**
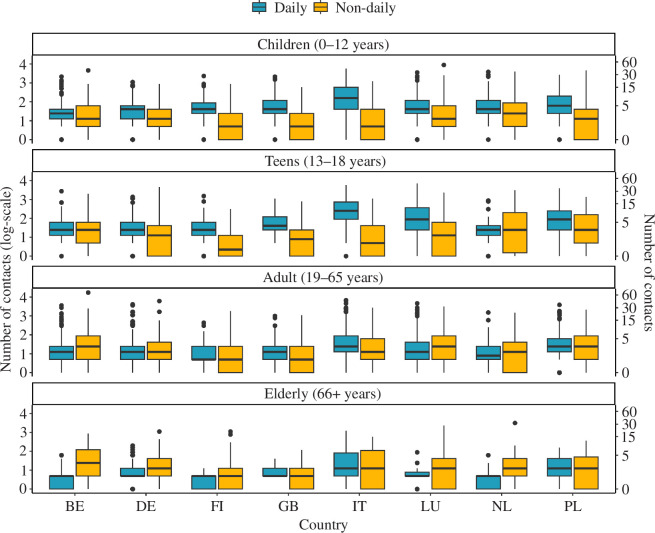
The distribution of the total number of daily and non-daily physical contacts from the POLYMOD study (Belgium (BE), Germany (DE), Finland (FI), Great Britain (GB), Italy (IT), Luxembourg (LU), The Netherlands (NL), and Poland (PL)).

The proportions of daily and non-daily physical contacts for the pre-pandemic and pandemic scenarios are presented in figure 1 and the electronic supplementary material, figure S3, respectively. For the pandemic scenario, correcting for under-reporting owing to fatigue increases the average number of reported contacts by 8% (electronic supplementary material, figure S1). In the former case, we first noticed that age affects the frequency of contact. In most of the countries considered, children and teens reported more daily than non-daily contacts, while adults and the elderly reported more non-daily contacts.

**Figure 2.**
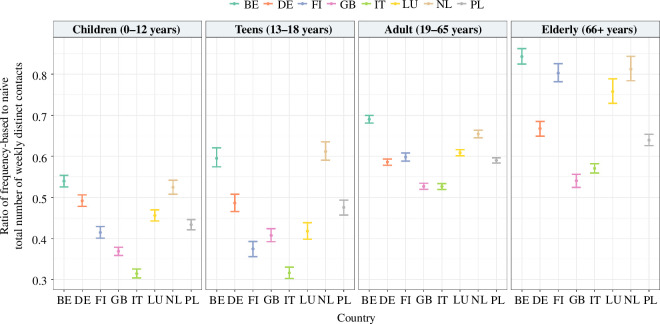
The ratio between frequency-based and naively calculated distinct contacts over a week in a pre-pandemic scenario, together with its 95% confidence interval obtained by a non-parametric bootstrap for children, teens, adults and the elderly in different countries.

Following the methodology presented in §2a, we calculated the total number of distinct contacts using the naive and frequency-based approaches for different European countries. Failure to account for repetition results in an overestimation of the total number of distinct contacts in a week, with an average overestimation of up to 45% (figure 2; electronic supplementary material, table S3). This pattern holds across different age groups and countries. Finland indicates a considerable difference in the ratio of frequency-based to naive total number of weekly distinct contacts between children (0.414, 95% confidence interval (CI): [0.401–0.429]) and elderly (0.802, 95% CI: [0.781–0.825]), while the Great Britain shows the smallest discrepancy, with only 17.2% difference between children and elderly. The difference between these two ratios decreases as age increases. Children show the most noticeable difference, with an average ratio of around 0.44 across countries. This discrepancy gradually reduces to about 0.46 for teens and further to 0.59 for adults. By contrast, the elderly display the least disparity, with an average ratio of 0.70 across countries. We further notice that the relative difference between the total number of contacts made by specific age groups varies depending on the approach considered (physical/all contacts) to reconstruct contact patterns, possibly affecting the contribution of such age groups in transmission (electronic supplementary material, figure S4).

#### (ii) Outbreak characteristics

**Figure 3.**
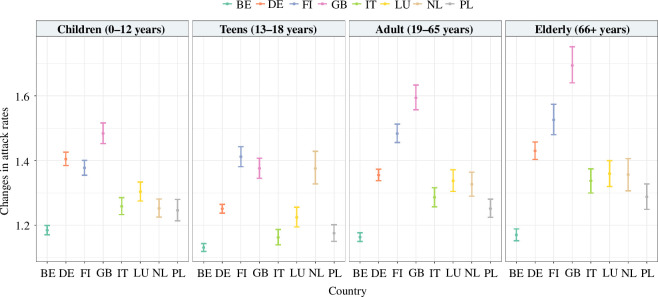
Changes in epidemic attack rates in influenza-like illness when simulating epidemics using a frequency-based approach compared to a naive approach during a pre-pandemic scenario, along with non-parametric bootstrapping for 95% confidence intervals.

Results of the simulation study suggest that epidemics are affected by the contact pattern considered over time (electronic supplementary material, table S4). Great Britain exhibits the highest discrepancy between the attack rates of the elderly and teens, with a difference of approximately 14% (elderly: 0.590, 95% CI: [0.571 – 0.608]; teens: 0.727, 95% CI: [0.709–0.744]). Consistently, higher attack rates were observed when assuming a naive reconstruction (refer to figure 3; electronic supplementary material, figure S5), especially at lower reproductive numbers, which resulted in a 33 and 5% average correction in attack rates for influenza-like disease and COVID-19-like disease, respectively. Furthermore, the frequency-based approach consistently results in a lower peak incidence and a longer time to peak (electronic supplementary material, figure S6). Regarding the overall epidemic duration, the difference is not apparent between the frequency-based and naive approaches for influenza-like illness, but a slight discrepancy is observed for COVID-19-like illness, with the former leading to epidemics with longer duration. In addition, a higher extinction rate is also observed when the frequency-based approach is considered (electronic supplementary material, table S5), with an increase ranging from 7.5% in Poland to 15.5% in Denmark.

### (b) Pandemic setting

#### (i) Contact repetition

In addition to investigating the impact of temporal reconstruction across different countries in Europe, we further describe how such contact profiles vary during a pandemic setting, by using the Belgian CoMix data as a case study. Different behaviours can be seen during the pandemic scenario, where for all age categories, the majority of reported contacts are daily contacts (electronic supplementary material, figure S3). As expected, we observed that the number of distinct contacts is greatly reduced when comparing it with the number of contacts during the pre-pandemic scenario. This holds for all contacts and only physical contacts. The overestimation of the total number of distinct contacts in a week between frequency-based and naive approaches during the pandemic was greater (77%) than in the pre-pandemic period with the decrease varying across age categories. Additionally, we observed higher differences between distinct contacts reconstructed for children and teens compared with adults and the elderly, with an average of 79 and 73%, respectively (electronic supplementary material, figure S7). While for adults and the elderly, the total number of distinct contacts seems to be stable over the different waves, a higher variation is observed for children and teens (electronic supplementary material, figure S8).

#### (ii) Outbreak characteristics

Considering the same respiratory infections assumed previously, we simulate epidemics during a pandemic scenario. Calculating attack rates using the frequency-based approach results in an underestimation, ranging from approximately 25% to 45% in influenza-like scenarios to about 60–85% in COVID-19-like scenarios when compared with the naive approach (electronic supplementary material, figure S9). This effect is also more pronounced in survey waves with stricter interventions (wave 19 compared with wave 43). When evaluating other epidemiological measures, we observed a lower peak incidence when simulating the epidemic with a frequency-based approach in the pandemic scenario (electronic supplementary material, figure S10), while the impact on the epidemic duration and time to peak was less pronounced in the pre-pandemic scenario and extended over a longer period in the pandemic scenario. Lastly, extinction rates depend on the reproduction number assumed but are always computed to be higher for the epidemics simulated with the naive approach, showing more pronounced relative differences in the pandemic scenario (electronic supplementary material, table S6).

### (c) Influential factors

**Figure 4.**
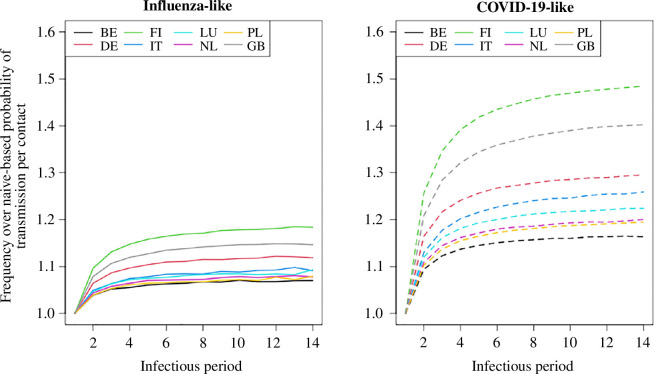
Comparison of the probability of infection per contact between frequency-based versus naive approach for Belgium with various infectious periods.

We examined mixing patterns with respect to intimacy level (electronic supplementary material, figure S11) and found more pronounced differences in the total number of distinct contacts when specifically considering physical interactions (45%), compared to analysing all contacts (39%). Furthermore, we consider the location where contacts occur by comparing interactions that take place within and outside the household setting. It can be seen that during the pre-pandemic period, the majority of contacts occurred outside the household (electronic supplementary material, figures S12 and S13), while during the pandemic, household interactions were the main components of mixing patterns. In particular, we observed that children and teenagers show high variability in terms of the number of contacts outside the home during the pandemic, possibly caused by the interventions in place. We examined the impact of temporal reconstruction on transmission dynamics across various lengths of infection periods, and as expected, the underestimation of the probability of infection per contact was smaller for shorter infection periods and became more significant for longer durations (figure 4). There is no difference in the probability of transmission per contact for a one-day infectious period, as it indirectly implies no repetition of contact during infection. The underestimation is observable in both diseases. However, its impact varies, with a more pronounced effect observed in the COVID-19-like disease in comparison with the influenza-like disease, while keeping the average number of contacts the same. Similar conclusions arise when comparing attack rates for Belgium using the pre-pandemic mixing patterns, with the difference between the frequency-based approach and the naive approach decreasing as the period of infection decreases (electronic supplementary material, table S7). Specifically, the mean differences in attack rates were 90, 87 and 86% for infection periods of 2, 5 and 7 days, respectively, in cases of influenza-like illness across age groups. We further investigated the impact of simulating the epidemic by adjusting the probability of transmission per contact to achieve the same reproduction numbers within the pre-pandemic scenario. We consistently observed an underestimation in the probability of transmission per contact across all countries when contact repetition is not considered (electronic supplementary material, table S8). This underestimation was more pronounced at higher reproduction numbers, ranging from 15% to 45%, with an average of 26%. Conversely, lower reproduction numbers indicated less underestimation, ranging from 6% to 23%, with an average of 10% (electronic supplementary material, figure S14). No major differences were observed when comparing the characteristics of outbreaks with constant reproductive numbers (electronic supplementary material, figures S15 and S16), except for Finland which exhibited slightly larger differences in the elderly (up to 20%).

## 4. Discussion

Social interactions are fundamental for characterizing and describing the transmission of respiratory infectious diseases, constituting a key element in modelling transmission dynamics. Population-level epidemic models often neglect the temporal component of human contact behaviour, treating interactions occurring on a specific day as independent from those on other days. In this study, we reconstructed contact interactions over the course of one week and investigated the implications of such a reconstruction in modelling the transmission of respiratory diseases. In particular, we considered two epidemiological settings in which human interactions have been shown to differ widely, i.e. during pre-pandemic and pandemic stages.

Our findings align with established evidence from other studies regarding the heterogeneity of contact behaviour, such as age, intimacy level, contact location and country [5,12,19,29,30]. We further demonstrated a high proportion of contact repetition, suggesting its influence on contact patterns over time. Considering the repetition of contact interactions, especially in epidemiological contexts characterized by a high frequency of contact repetition, such as schools and workplaces, could provide valuable insights to develop intervention strategies in these locations. Furthermore, we observed an increase in the proportion of daily physical contacts compared with all contacts. This holds alongside a decrease in the total number of contacts during the COVID-19 pandemic, probably attributable to the non-pharmaceutical interventions implemented during the pandemic time [9]. This finding can provide useful insights into setting population-level interventions for future epidemic or pandemic threats and further underlines the importance of collecting comprehensive contact data in various settings, ensuring a better understanding of different human interactions. One of the key elements that characterize super-spreading events is a particularly high number of interactions [31,32]. In the attempt to gain insights into the drivers of super-spreading events, one could reconstruct the number of distinct contacts made by specific groups of individuals in specific locations. Our study highlights the significance of accounting for the repetition of contact behaviour, specifically addressing the potential repetition of contacts. Linked with viral shedding kinetics, environmental factors and other contact characteristics (e.g. duration) [28], the total number of distinct contacts provides crucial insights for identifying the circumstances under which superspreading events can take place.

To unravel the implications of accounting for temporal contact patterns in modelling, we simulated epidemics by implementing both a contact structure that accounts for repetition, i.e. frequency-based approach, and a contact structure in which contact behaviour is considered independent during consecutive days, i.e. naive approach. The results indicate that omitting the temporal component of contact behaviour might lead to an underestimation of the transmission parameter [17], and this becomes particularly noticeable with an increase in contact repetitions, typical of the pandemic period or in diseases with longer infectious periods. While the pattern remained similar across countries, the magnitude of the effect varied owing to differing contact patterns. Moreover, we investigated other outbreak characteristics (e.g. time to peak, peak incidence and epidemic durations), showing an impact of accounting for the repetition of contacts over time. Furthermore, we demonstrate that adjusting the probability of transmission in the frequency-based approach to achieve equal reproduction numbers as in the naive approach leads to similar outbreak characteristics. This holds true across all countries except Finland, where attack rates for the elderly are slightly below 1. This is owing to the fact that the attack rate in Finland for the elderly is quite low (10.6% for the naive approach and 13.2% for the frequency-based approach), but further investigation is warranted. While outbreak characteristics are shown to depend on the repetition of contact interaction, we observed that accounting for repetition has a lower impact on infections with high transmissibility, in line with what has been previously observed by Smieszek *et al.* [21]. Therefore, it is crucial to acknowledge that, apart from contact patterns and transmission probability, other characteristics (e.g. infectious period distribution) also play a role in shaping the epidemiological outcome of an epidemic [33].

Social contacts exhibit greater complexity than the model assumptions. Specifically, our assumption of daily contact, where an individual interacts every day with the same person, may lead to an overestimation of repetition. By contrast, our assumption of non-daily contact, occurring each time with a different individual, may underestimate the impact of repetition. However, we focused on a time frame of one week, where such assumptions are supposed to have a limited effect on the overall results. Additionally, we only accounted for contact reciprocity at the population level rather than the individual level (e.g. if is a daily contact of *j*, this does not imply that *j* is a daily contact of *i*) [17]. Further investigations are needed to assess whether individual reciprocity may have additional effects. We also did not account for other contact components, such as repeated contacts within a day, which could affect transmission probability. Future research could explore the impact of repeated contacts within a day on disease transmission dynamics, by incorporating time-used data or data from wearable proximity sensors to capture more detailed contact patterns. Furthermore, we restricted our analysis to the stochastic S-I-R epidemic model. While it is possible to extend this to more complex models, we expect that the general findings established in this article will similarly apply to other models and modelling frameworks [21]. Finally, CoMix is a longitudinal survey where participants may experience fatigue as the survey progresses [2]. When correcting for the under-reporting, we implicitly assume that the impact of fatigue is consistent for both daily and non-daily contacts. However, it can be argued that this effect might vary across different contact frequencies. One approach could be to ask about daily contacts once and only ask about non-daily contacts in later survey waves, as a way to minimize under-reporting while completing the survey.

## 5. Conclusions

This study describes the frequency of social interactions responsible for the transmission of respiratory infectious diseases, in several European Union countries in a pre-pandemic setting and during the COVID-19 pandemic in Belgium. Our findings indicate the importance of considering contact repetition in social contact data when assessing the total number of infections attributable to an infectious case. Additionally, our study suggests the significance of investigating the temporal component in population-level models for the spread of infectious diseases. Failure to account for the repetition of contacts may result in underestimating the transmission potential of infectious diseases. Moreover, our analysis reveals that mixing patterns are contingent upon individual characteristics, such as age and specific locations, including households. These insights provide valuable guidance for shaping both local and global intervention measures to limit the spread of respiratory infections.

## Data Availability

The POLYMOD data and the Belgium CoMix data are available from Mossong et al. [2008]: (doi: 10.5281/zenodo.3746312) [5] and Coletti et al. [2020]: (doi: 10.5281/zenodo.10549953) [10], respectively. The source code for this project is also available at Zenodo (doi:10.5281/zenodo.12607242) [34]. Supplementary material is available online (doi:10.6084/m9.figshare.c.7356976) [35].
